# The impact of complex karyotype on the overall survival of patients with relapsed chronic lymphocytic leukemia treated with idelalisib plus rituximab

**DOI:** 10.1038/s41375-019-0533-6

**Published:** 2019-08-19

**Authors:** Karl-Anton Kreuzer, Richard R. Furman, Stephan Stilgenbauer, Ronald L. Dubowy, Yeonhee Kim, Veerendra Munugalavadla, Esther Lilienweiss, Hans Christian Reinhardt, Paula Cramer, Barbara Eichhorst, Peter Hillmen, Susan M. O’Brien, Andrew R. Pettitt, Michael Hallek

**Affiliations:** 1 Department I of Internal Medicine, University of Cologne, Cologne, Germany; 2 Weill Cornell Medical College, New York, NY USA; 3 Department III of Internal Medicine, Ulm University Medical Center, Ulm, Germany; 4 Gilead Sciences, Inc., Foster City, CA USA; 5 Center for Molecular Medicine Cologne, and Cologne Excellence Cluster on Cellular Stress Responses in Aging-Associated Diseases (CECAD), University of Cologne, Cologne, Germany; 6 St. James’s University Hospital, Leeds, UK; 7 University of California-Irvine, Irvine Chao Family Comprehensive Cancer Center, Orange, CA USA; 8 University of Liverpool, Liverpool, UK

**Keywords:** Cancer genetics, Cytogenetics, Chronic lymphocytic leukaemia

## To the Editor

Relapsed or refractory (R/R) chronic lymphocytic leukemia (CLL) is frequently associated with the acquisition or enrichment of chromosomal and molecular genetic features. These include deletion of the short arm of chromosome 17 (del[17p]), mutations in the tumor-suppressor protein p53 gene (*TP53*), lack of somatic mutations in the variable region of the immunoglobulin heavy chain, and others [1–4]. Complex karyotype (CK) is defined as at least three distinct chromosomal abnormalities present in more than one metaphase [5]. The presence of CK abnormalities is an adverse prognostic factor and associated with inferior outcomes in patients with CLL after treatment with chemotherapies and targeted therapies [6–12].

Idelalisib—a selective inhibitor of phosphatidylinositol-3-kinase delta—in combination with rituximab led to significant prolongation of progression-free survival (PFS) and overall survival (OS) compared with that seen with rituximab plus placebo in patients with relapsed CLL and significant comorbidities in a randomized, double-blind, phase 3 study (NCT01539512; the primary study) [13]. Patients who progressed on the primary study could enroll in an extension study (NCT01539291) to receive idelalisib monotherapy [13]. The primary study was terminated prematurely due to the superior efficacy of idelalisib/rituximab combination; patients still on treatment could also enroll in the extension study. In this exploratory analysis, we examined the clinical outcomes of idelalisib-treated patients enrolled in the above-mentioned studies with or without CK, as determined by peripheral blood lymphocyte karyotyping.

From May 2012 to August 2013, 220 eligible patients (Table S1) were randomly assigned to idelalisib/rituximab (*N* = 110) or treatment with placebo/rituximab (*N* = 110) in the primary study [13]. Overall, 161 patients enrolled in the extension study initiated in October 2012. Samples for metaphase spreads were obtained from all patients and were processed (supplemental text) in two different laboratories (NJ, USA; and Cologne, Germany). The samples processed in the laboratory in Cologne had a karyotypic success rate of 99%. The samples from the US sites were processed at the US laboratory and were subsequently sent to Germany for the karyotype analysis. Approximately half of the US samples contained very few metaphases or metaphases with poor quality; hence, karyotype analyses were performed successfully in only 51% of samples from the American sites. Of the 220 patients randomized in the primary study, successful stimulated karyotypes were obtained from 127 patients; 63/110 (57%) in the idelalisib arm and 57/110 (52%) in the placebo arm, with an overall karyotypic success rate of 55% (Fig. S1). The proportion of CK-positive and CK-negative patients was comparable between treatment arms; 26/63 (41%) patients in the idelalisib arm and 24/57 (42%) patients in the placebo arm were CK-positive (*p* = 1.000; Fig. S1). A listing of patients’ karyotypes is provided in Table S2.

Demographic and baseline characteristics and prognostic disease parameters for patients with successful karyotyping, summarized in Table S3, were mostly balanced between the CK and non-CK groups within each arm. Regardless of CK status, most patients were male. Median age of patients with and without CK was 69 and 73 years, respectively. All patient subgroups were pretreated with a median of 3–3.5 prior therapies. More than 80% of the patients had anemia, thrombocytopenia, or neutropenia of any grade at baseline. A higher percentage of patients with CK (62%) also had del[17p] and/or *TP53* mutation compared with patients without CK (43%; Table S3), although 38% of patients with CK did not exhibit *TP53* aberrations. Most patients had a high or very high CLL-International Prognostic Index risk score and a higher proportion of patients with CK were in the “very high risk” group (Table S3). Demographic and baseline characteristics were balanced between successfully karyotyped patients and those who were not successfully karyotyped (Table S4).

As of the August 16, 2018, final cutoff, the median (range) follow-up for the successfully karyotyped patients in the idelalisib/rituximab arm was 29.2 (0.3, 67.6) months. In patients treated with idelalisib/rituximab, the overall response rates for CK-positive and -negative groups were 81% and 89%, respectively (odds ratio 0.5, *p* = 0.3509; Table 1); all were partial responses.

**Table 1:** Best overall response rate in the successfully karyotyped patients treated with idelalisib/rituximab per IRC assessment

	Idelalisib+rituximab	
	CK-positive, *N* = 26	CK-negative, *N* = 37
ORR, *n* (%)^a^	21 (80.8)	33 (89.2)
95% CI^b^	60.6, 93.4	74.6, 97.0
Complete response	0	0
Partial response	21 (80.8)	33 (89.2)
Stable disease	3 (11.5)	2 (5.4)
Progressive disease	1 (3.8)	0
Not evaluable	1 (3.8)	2 (5.4)
Odds ratio for ORR^c^	0.5	
95% CI for odds ratio	0.1, 2.1	
* p*-value	0.3509	

CI confidence interval, CK complex karyotype, IRC Independent Review Committee, ORR overall response rate

^a^ORR is the percentage of patients who had best overall response of complete response or partial response

^b^95% CI for ORR is based on the exact method

^c^Odds ratio and 95% CI are calculated without any adjustment

**Fig. 1:**
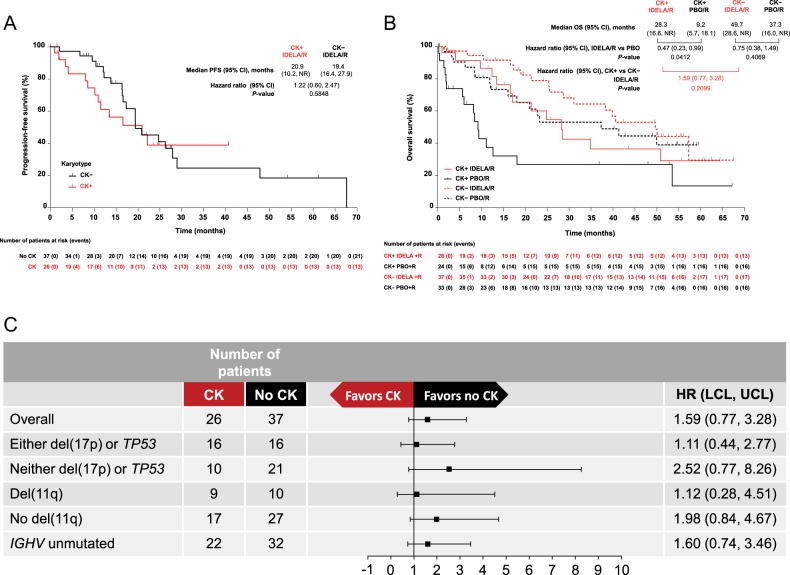
**a** Progression-free survival in patients with successful karyotyping in the idelalisib arm. **b** Overall survival in patients with successful karyotyping. **c** Forest plot of hazard ratios for OS by prespecified subgroups and the presence or absence of complex karyotype in the idelalisib arm. CI confidence interval, CK complex karyotype, HR hazard ratio, IDELA idelalisib, IGHV immunoglobulin heavy-chain variable region gene, LCL lower confidence limit, no CK no complex karyotype, NR not reached, OS overall survival, PBO placebo, PFS progression-free survival, R rituximab, UCL upper confidence limit

PFS was comparable for patients treated with idelalisib/rituximab, independent of the presence or absence of CK (Fig. 1a). In the CK-positive and CK-negative groups, median (Q1, Q3) PFS was 20.9 (8.5, not reached [NR]) months and 19.4 (16.4, 28.9) months, respectively. The unadjusted hazard ratio (HR) (95% confidence interval [CI]) for the difference between CK-positive vs CK-negative was 1.22 (0.60, 2.47; *p* = 0.5848).

Median (range) follow-up for CK-positive patients in the idelalisib/rituximab arm was 16.8 (1.0, 64.4) months and 7.4 (0.2, 67.2) months for those in the placebo/rituximab arm. With the two treatment groups combined, the median (range) follow-up for CK-positive patients was 11.4 (0.2, 67.2) months. Among CK-positive patients, death occurred in 13/26 (50%) patients treated with idelalisib/rituximab and in 16/24 (66.7%) patients treated with placebo/rituximab. Median (Q1, Q3) OS seemed longer in the CK-positive group treated with idelalisib (28.3 [16.6, NR] months), compared with 9.2 (2.0, 53.5) months in CK-positive patients who received placebo/rituximab (Fig. 1b). The unadjusted HR (95% CI) of 0.47 (0.23, 0.99; *p* = 0.0412) showed favorable effect on OS with the idelalisib treatment. No significant difference in OS was noted between patients with or without CK treated with idelalisib/rituximab. Median (Q1, Q3) OS was 28.3 (16.6, NR) and 49.7 (25.5, NR) months for the CK-positive and CK-negative group, respectively, with unadjusted HR (95% CI) of 1.59 (0.77, 3.28) and *p* = 0.2099 (Fig. 1b). Co-presence of CK and del[17p], *TP53* mutation, or del[11q] did not significantly affect OS in patients treated with idelalisib/rituximab (Fig. 1c). There were no differences in PFS and OS between patients with vs without successful karyotyping (Table S5).

In this study, peripheral stimulated lymphocyte karyotyping was performed in the context of a multicenter, international, randomized trial. Our analysis suggests that CK-positive patients treated with idelalisib/rituximab did not exhibit a significantly shortened survival compared with those who were CK-negative. In addition, the primary beneficial effect of adding idelalisib to rituximab treatment in R/R CLL patients with CK was reflected in OS prolongation compared to those who received only rituximab.

The deleterious impact of the presence of CK on clinical outcomes in patients with R/R CLL after treatment with various chemotherapeutic regimens or targeted therapies has been well documented [6–12]. Interestingly, long-term follow-up studies of patients receiving ibrutinib have shown varying results. Thompson et al. reported that in 88 R/R CLL patients treated with ibrutinib, after 3 years of follow-up, CK was associated with shorter OS in both univariate (25 months vs NR; *p* = 0.007) and multivariate analyses (HR [95% CI], 5.9 [1.6–22.2], *p* = 0.008). In addition, the survival of patients with CK was significantly inferior (*p* = 0.02) to the survival of patients without CK [9]. In a 5-year follow-up of 132 patients treated with ibrutinib in a phase 1b/2 study, median PFS and OS were shorter for those with CK vs those without. Of interest, when these results were further stratified by the presence or absence of del[17p], the median PFS and OS of patients with CK without del[17p] were considerably longer than of those with del[17p]. Thus, after multivariate analyses, the presence of CK was no longer significantly associated with PFS or OS, while the presence of del[17p] remained so [14]. In contrast, after a median follow-up of 19 months, no significant differences were observed in PFS or OS in ibrutinib-treated patients with or without CK in a randomized phase 3 study [15]. In this study, CK data were missing from 22% of patients and the authors did recognize that the relatively short follow-up time and incomplete data limited the interpretability of their results.

Overall, the data presented here suggest a beneficial effect of idelalisib/rituximab vs rituximab alone on OS regardless of CK status, even among patients who presented with del[17p] or *TP53* mutation. However, owing to the small sample size employed for this post hoc analysis and the possibility of competing causes of death from idelalisib-related toxicities, these results should be re-evaluated in a larger patient population. Another limitation of this study is that the quality of the metaphase harvests was inconsistent, since two different laboratories were used, and methodologies were not sufficiently harmonized as reflected by the different success rates of the two laboratories. However, no differences in PFS and OS were seen between patients with vs without successful karyotyping when treated with idelalisib/rituximab.

Our results, along with those presented for other targeted therapies, indicate that further prospective, larger clinical studies are needed to guide individualized treatment decisions in patients with R/R CLL and CK and provide guidance on treatment sequencing. In addition, it may be worthwhile to consider chromosome banding as an additional prognostic risk factor.

## Supplementary information

Supplemental Materials
