# Supplementary material for: The impact of complex karyotype on the overall survival of patients with relapsed chronic lymphocytic leukemia treated with idelalisib plus rituximab
Source: Leukemia. 2019 Aug 19;34(1):296–300. doi: 10.1038/s41375-019-0533-6 (PMC7214265; doi:10.1038/s41375-019-0533-6)
Supplement: Supplementary file 1 — Supplemental Materials [file 41375_2019_533_MOESM1_ESM.docx]

**Supplementary Information**

**The Impact of Complex Karyotype on the Overall Survival of Patients with Relapsed Chronic Lymphocytic Leukemia Treated with Idelalisib Plus Rituximab**

Karl-Anton Kreuzer^1^, Richard R. Furman^2^, Stephan Stilgenbauer^3^, Ronald L. Dubowy^4^, Yeonhee Kim^4^, Veerendra Munugalavadla^4^, Esther Lilienweiss^1^, Hans Christian Reinhardt^1,5^, Paula Cramer^1^, Barbara Eichhorst^1^, Peter Hillmen^6^, Susan M. O’Brien^7^, Andrew R. Pettitt^8^, and Michael Hallek^1,5^

^1^Department I of Internal Medicine, University at Cologne, Cologne, Germany

^2^Weill Cornell Medical College, New York, NY, USA

^3^Department III of Internal Medicine, Ulm University Medical Center, Ulm, Germany

^4^Gilead Sciences, Inc., Foster City, CA, USA (at the time the study was conducted, and manuscript drafted)

^5^Center for Molecular Medicine Cologne, and Cologne Excellence Cluster on Cellular Stress Responses in Aging-Associated Diseases (CECAD), University of Cologne, Cologne, Germany

^6^St. James's University Hospital, Leeds, UK

^7^University of California-Irvine, Irvine Chao Family Comprehensive Cancer Center, Orange, CA, USA

^8^University of Liverpool, Liverpool, UK

**Table S1.** Key eligibility criteria

**Table S2.** Karyotypes identified in patients for whom karyotyping was successful

**Table S3.** Demographic and baseline characteristics in patients with successful karyotyping

**Table S4.** Demographic and baseline characteristic of patients who were successfully karyotyped vs not successfully karyotyped

**Table S5.** Overall survival and progression-free survival in patients who were successfully karyotyped vs not successfully karyotyped

**Figure S1.** Consort diagram. CK, complex karyotype; IDELA, idelalisib; PBO, placebo; R, rituximab.

**Subjects and Methods**

*Study design and treatments*

The primary study (NCT01539512) was a randomized, double-blind, placebo-controlled, phase 3 trial evaluating the addition of idelalisib to rituximab in patients with relapsed/refractory chronic lymphocytic leukemia (CLL) and significant comorbidities. The details of study design and treatment were reported previously (1). Briefly, randomization was stratified by the presence of del(17p) and/or*TP53* mutation and immunoglobulin heavy chain mutation status. Patients were randomized to receive either idelalisib 150 mg twice daily or placebo, administered in combination with an intravenous rituximab 375 mg/m^2^ on day 1, week 0, followed by 500 mg/m^2^ every 2 weeks for four doses and then every 4 weeks for three doses, for a total of eight infusions. Treatments were administered until disease progression, unacceptable toxicity, or study discontinuation (1).

Patients who experienced progressive disease (PD) on the primary study could enroll into an extension study (NCT01539291) in which patients originally randomized to placebo/rituximab received oral idelalisib 150 mg twice daily, while those originally randomized to idelalisib/rituximab received idelalisib 300 mg twice daily. The primary study was unblinded and terminated early on the recommendation of the Data Monitoring Committee due to overwhelming difference in the efficacy between the idelalisib and control arms. After primary study unblinding, all patients remaining on the primary study could enroll into the extension study to receive an open-label single-agent idelalisib 150 mg twice daily.

All study protocols were approved by the institutional review board or independent ethics committee at each participating center. Studies were conducted in accordance with the Declaration of Helsinki and the International Conference on Harmonisation Guidelines for Good Clinical Practice.

*Patients*

Eligible for enrollment were patients with recurrent, previously treated CLL with measurable lymphadenopathy, who experienced PD <24 months since the last line of therapy, were unfit for cytotoxic therapies, and had at least two prior cytotoxic regimens or one prior treatment with an anti-CD20 antibody (1). Patients with confirmed malignant transformation from CLL to an aggressive lymphoma were excluded. The key inclusion and exclusion criteria are presented in **Supplemental** **Table 1**. All patients provided signed informed consent prior to participating in the study.

*Objectives*

The primary endpoint of the randomized study was progression-free survival (PFS); key secondary endpoints included overall response rate (ORR) and overall survival (OS) (1). This retrospective exploratory analysis was conducted to evaluate the impact of the presence or absence of complex karyotype (CK) on PFS, ORR, and OS in patients treated with idelalisib plus rituximab, and the effect of treatment with idelalisib/rituximab vs placebo/rituximab on OS in the CK-positive group, using the data from the both primary and extension studies.

*Assessments*

Computer tomography scans or magnetic resonance imaging obtained every 8 to 12 weeks were evaluated by an Independent Review Committee (IRC) to assess response and disease progression using standardized International Workshop on Chronic Lymphocytic Leukemia criteria (2). An IRC included a board-certified radiologist and an independent board-certified hematologist or oncologist who performed independent reviews of response and disease progression for each patient.

Metaphase preparations of fresh peripheral blood samples were done in two different central laboratories; a commercial laboratory in the US (Rutherford, New Jersey, USA) processed samples from US sites, and the German CLL study group laboratory (Cologne, Germany) processed samples from European sites. Following the initial metaphase preparation at the two sites, all karyotypic analyses were completed in the Cologne laboratory. Chromosome banding analyses were performed following interleukin (IL)-2/CpG-stimulated culture (3) of peripheral blood lymphocytes (48 hours with tetradecanoyl-phorbol acetate, and 72 hours with CpG-oligodeoxynucleotides and IL-2), and karyotypes were analyzed per the International System for Human Cytogenetic Nomenclature, 2013 (4, 5). If an aberrant karyotype was detected, a minimum of 12 additional metaphases were analyzed when available. Chromosomal aberrations were considered clonal if they occurred in ≥3 metaphases. If available, at least 20 metaphases in a sample were investigated to confirm the absences of any chromosomal aberrations. Del(17p) and del(11q) were determined by fluorescent in situ hybridization on the interphase peripheral blood leukocytes, and *TP53* mutation was assessed by standard Sanger sequencing (German CLL Study Group Laboratory, Ulm, Germany) (6).

*Statistical analyses*

All the exploratory analyses were performed in successfully karyotyped patients. PFS was defined as the interval from randomization to the first documentation of definitive PD per IRC or death on study. Definitive PD was defined as CLL progression based on standard criteria other than lymphocytosis alone (2, 7). OS was defined as the interval from randomization to death from any cause and analyzed without any adjustment for treatment switching. PFS and OS were estimated using Kaplan-Meier methods. Hazard ratios and 95% confidence intervals (CI) were calculated using Cox proportional hazards regression model. ORR was defined as the proportion of patients who achieved a complete response or partial response based on the International Workshop on Chronic Lymphocytic Leukemia criteria assessed by IRC (2). Odds ratios and the corresponding 95% CIs were presented. The analyses were performed using data from both the primary and extension studies with the cutoff date of May 2, 2016. PFS, ORR, and OS were compared between CK-positive and -negative groups for patients randomized to the idelalisib arm. OS was further compared by treatment arms using unadjusted intent-to-treat analysis in the CK-positive group.

Analyses were performed using SAS, version 9.4. The study was not designed and powered to detect statistical differences between CK-positive and -negative groups. Given the exploratory nature of the analyses, no adjustment was made for multiple comparisons and a nominal *p*-value <0.05 was considered as statistically significant.

**Table S1.** Key eligibility criteria

| **Inclusion criteria** | |
| --- | --- |
| Relapsed CLL | CLL progression <24 months since last therapy  Treatment warranted according to IWCLL criteria |
| Lymphadenopathy | Presence of ≥1 measurable nodal lesion |
| Prior therapies | ≥1 anti-CD20 antibody-containing therapy or ≥2 prior cytotoxic therapies |
| Appropriate for noncytotoxic therapy | CIRS score >6, CrCl ≥30–<60 mL/min, baseline serum total bilirubin ≤1.5 × ULN (unless elevated due to Gilbert’s syndrome); serum ALT and AST ≤2.5 × ULN or grade 3/4 neutropenia or thrombocytopenia due to prior myelotoxicity |
| Bone marrow function | Any-grade anemia, neutropenia, or thrombocytopenia allowed |
| Karnofsky score | ≥40 |
| **Exclusion criteria** | |
| Transformation | Known histological transformation from CLL to an aggressive lymphoma |
| Infection | Evidence of ongoing systemic bacterial, fungal, or viral infection at the time of the start of study treatment |

ALT, alanine aminotransferase; AST, aspartate aminotransferase; CIRS, Cumulative Illness Rating Scale; CLL, chronic lymphocytic leukemia; CrCl, creatinine clearance; IWCLL, International Workshop on Chronic Lymphocytic Leukemia; ULN, upper limit of normal.

**Table S2.** Karyotypes identified in patients for whom karyotyping was successful

| **#** | **Karyotype** |
| --- | --- |
| **Idelalisib/rituximab** | |
|  | ***No complex karyotype*** |
| 1 | 45,XY,der(17;20)(q10;q10)[7]/46,XY[6] |
| 2 | 48,XX,+12,+19[14] |
| 3 | 46,XY,del(6)(q21q23)[17] |
| 4 | 46,XX[22] |
| 5 | 45,XY,-8,der(17)t(8;17)(q11;p13)[7]/46,XY[2] |
| 6 | 46,XY,t(10;13)(q22;q12)[16]/46,XY[4] |
| 7 | 46,XY,del(13)(q13q14)[3]/46,XY[17] |
| 8 | 46,XX,del(11)(q22q23)[9] |
| 9 | 46,XY,der(14)?del(14)(q24q24)[16] |
| 10 | 46,XY,inv(9)(p13q13)c,del(11)(q14q23)[7]/46,XY,inv(9)(p13q13)c[6] |
| 11 | 46,XX[20] |
| 12 | 46,XY,del(11)(q22q23),t(13;13)(q34;q14)[10] |
| 13 | 46,XY,t(16;17)(q2?2;?q11)[9] |
| 14 | 46,XY,der(17)?i(17)(q10)[5]/46,XY[2] |
| 15 | 46,XY,t(6;13)(p12;q12),del(17)(p11)[10] |
| 16 | 46,XX,t(3;9)(p21;q34),der(11)r(11;?)(p15q25;?)[5] |
| 17 | 46,XX[20] |
| 18 | 46,XY,del(11)(q22q23),del(13)(q12q14)[7] |
| 19 | 47,XX,t(8;16)(q13;p11),+12[11] |
| 20 | 46,XX,del(11)(q14q23)[3]/46,XX[1] |
| 21 | 46,XY,t(?8;14)(q24;q32)[4] |
| 22 | 46,XY,del(1)(q42)[9]/46,XY,del(1)(q42),del(11)(q22q23)[3]/46,XY[4] |
| 23 | 47,XX,+12[4] |
| 24 | 47,XY,+12[12] |
| 25 | 46,XY,del(11)(q14q23)[3]/46,XY,t(2;3)(q21;q21),del(11)(q14q23)[4]/46,XY[2] |
| 26 | 47,XY,+12[5]/47,XY,+12,del(13)(q12q14)[5] |
| 27 | 46,XY,del(11)(q22q23)[5]/46,XY[8] |
| 28 | 46,XY,add(8)(p?23),del(11)(q22q23)[3] |
| 29 | 46,XX,del(4)(p16)[3]/46,XX,del(4)(p16),del(5)(q11q14)[10] |
| 30 | 46,XY[20] |
| 31 | 47,XY,+12[7]/47,XY,+der(12)add(12)(q24)[4]/47,XY,+12,del(13)(q12q14)[2] |
| 32 | 47,XY,+12[10] |
| 33 | 46,XX,del(13)(q12q22)[13] |
| 34 | 46,XY,del(6)(q13q25)[12] |
| 35 | 47,XY,+12[10]/47,XY,+12,t(14;18)(q32;q21)[5] |
| 36 | 46,XY[20] |
| 37 | 47,XY,+12[10]/47,XY,add(4)(p16),+12[2] |
|  | ***Complex karyotype present*** |
| 1 | 44~46,XY,del(6)(q23q27),der(7)?add(7)(p21),der(17)t(17;?18)(p11;q11),add(22)(p11), inc[cp15] |
| 2 | 46,XY,t(13;17)(q12;p11)[2]/45,XY,del(6)(p21),-13,der(17)t(13;17)(q12;p11)[cp6]/46,XY[2] |
| 3 | 45,XY,der(8)?inv(8)(q13q24),del(10)(q22q24),der(13;20)(q10;q10),del(17)(p11)[12] |
| 4 | 43~45,XY,-X,der(1)?del(1)(p34),-6,-8,-9,-10,-11,+add(12)(p13),-13,-15,?+17,-20,+mar,inc[cp6] |
| 5 | 46,XY,del(5)(q?22),add(18)(p11),add(19)(?q13),inc[cp13]/46,XY[3] |
| 6 | 45~46,XX,del(3)(p13),add(4)(p16),der(7)?t(3;7)(q21;p22),-17,der(20)?t(17;20)(q21;q13),  add(21)(p11),+mar[cp13] |
| 7 | 42,XY,-2,-4,-9,der(11)t(11;?13)(q23;q21),der(12)t(?4;12)(q21;q24),-13,add(13)(q34),  der(15)t(2;15)(q21;q22),der(17)t(17;?)(p11;?),?t(17;18)(q12;q22)[cp8] |
| 8 | 45,XY,+der(12)t(12;?18)(q13;q11),-15,add(16)(p11),i(17)(q10),-18,der(21)t(?15;21) (q11;p11)[13] |
| 9 | 47,XY,del(6)(q13q25),del(9)(q13),+12,der(17)t(10;17)(q11;p11)[11] |
| 10 | 45,XY,del(10)(q24),del(11)(q22q23),-13,der(17)t(13;17)(q12;p11)[2]/45,XY,der(6)t(6;13) (p?23;q12),del(10)(q24),del(11)(q22q23),-13,del(17)(p11)[2]/46,XY[5] |
| 11 | 45,XY,add(3)(p25),add(5)(q35),add(7)(q36),del(11)(q14q23),add(15)(p11),-17[cp6] |
| 12 | 45,XY,add(3)(p13),-4,der(6)t(6;11)(q27;q12),-8,der(10)t(?8;10)(q13;q26),del(11)(q14q23)[cp7] |
| 13 | 44,XX,der(1)?del(1)(q41),add(4)(p16),der(6;13)(q10;q10),?der(15)t(15;17)(q11;q11),-17,-18, +mar[2]/45~46,XX,der(1)?del(1)(q41),add(4)(p16),?der(6)t(6;?),der(14)?ins(14;?)(q11;?),-17,-18,+mar1,+mar2[cp6]/46,XX[1] |
| 14 | 45,XY,del(11)(q22q23),del(13)(q14q14),der(14)?del(14)(q24q32),der(17;18)(q10;q10),inc[cp16] |
| 15 | 46,XX,del(13)(q12q14),del(14)(q24q32),i(17)(q10)[cp3]/45,XX,-13,del(14)(q24q32), der(17)t(13;17)(q13;p12)[2]/46,XX,del(14)(q24q32),del(17)(p13)[1] |
| 16 | 46,XY,del(6)(q21q23),-10,add(11)(q14),-13,del(13)(q12q21),der(18)t(18;?)(p11;?),+mar1,  +mar2[cp6]/46,Y,der(?X)?del(X)(p11),del(6)(q21q23),-10,add(12)(q24),-13,del(13)(q12q21),  der(18)t(18;?)(p11;?), mar1,+mar2[4] |
| 17 | 46,XY,der(2)t(2;13)(q21;q14),der(13)t(2;13)(q31;q14),i(18)(q10),der(21)?dup(21)(q11q22)[2]/  46,XY,der(2)t(2;13)(q21;q14),del(13)(q12q14),der(13)t(2;13)(q31;q14),i(18)(q10),  der(21)?dup(21)(q11q22)[5] |
| 18 | 45,XY,der(11)del(11)(q22q23)t(11;18)(q23;q21),del(13)(q14q22),-18[10] |
| 19 | 46,XY,del(11)(q22q23)[3]/46,XY,del(3)(p22),der(9)?del(9)(q31q34),del(11)(q22q23),add(12)(p13),add(17)(p11),add(21)(p11)[2]/46,XY[2] |
| 20 | 46,X,-Y,t(4;9)(q31;q13),+12,i(17)(q10)[17] |
| 21 | 46,XX,?+X,+3,del(3)(p21),del(3)(p21),der(9)t(9;17)(q22;q21),del(13)(q12q21),del(14)(q24),  add(15)(q22),-17,der(20)t(1;20)(p13;p13),+mar,inc[cp6] |
| 22 | 47,XY,del(11)(q14q23),+12,del(13)(q12q14)[11] |
| 23 | 46,XX,-6,der(?11)t(8;?11)(q11;q13),+12,-13,t(14;18)(q32;q21),+der(?19)t(13;?19)(q14;p13),  +der(?)t(?;11)(?;q13)[14] |
| 24 | 46,XY,+12,der(16)?del(16)(q?22),-17,add(18)(q23)[10] |
| 25 | 47,XY,+12,t(19;22)(q13;q12)[5]/47,XY,-11,+12,t(19;22)(q13;q12),+mar[10]/46,XY[3] |
| 26 | 45,XX,-6,del(11)(q22q23),add(19)(p13)[2]/46,XX[18] |
| **Placebo/rituximab** | |
|  | ***No complex karyotype*** |
| 1 | 45,XX,-16,der(17)t(17;?18)(p11;q11),-18,-20,inc[cp5] |
| 2 | 46,XY,t(9;13)(q22;q12)[9]/46,XY,t(8;17)(q23;q12),t(9;13)(q22;q12)[3] |
| 3 | 46,XX,t(9;13)(q13;q12)[17] |
| 4 | 45~46,XY,t(1;6)(q21;q21),der(4)t(4;17)(p16;q21),-10,-17,-18,-21[cp8] |
| 5 | 44~45,XX,-3,-8,-10,-14,der(17)t(8;17)(q13;p11)[cp10] |
| 6 | 46,XX,i(17)(q10)[4] |
| 7 | 46,XY,del(11)(q22q23)[3] |
| 8 | 47,XX,del(6)(q12),del(11)(q22q23)[cp4]/46,XX[3] |
| 9 | 45,XY,der(17)t(17;18)(p11;q11),-18[3]/46,XY,del(6)(q13q23),del(10)(q22q25)[3]/ 46,XY,add(6)(p25)[2] |
| 10 | 46,XX,del(11)(q13q23)[4]/46,XX,del(11)(q13q23),del(13)(q12q14)[6] |
| 11 | 46,XY,add(17)(p11)[cp4]/46,XY,del(17)(p11)[cp3]/46,XY[11] |
| 12 | 45,XY,der(8;17)(q10;q10),del(11)(q14q23)[8] |
| 13 | 46,XX,t(4;14)(q21;q32),del(13)(q12q21)[7]/46,XX[3] |
| 14 | 46,XX,del(1)(q42)[8]/46,XX[6] |
| 15 | 46,XY,del(14)(q24)[12] |
| 16 | 47,XX,del(11)(q14q25),+12[3]/47,XX,+12[18] |
| 17 | 46,XY,der(4)?del(4)(p14),del(11)(q14q23)[cp7] |
| 18 | 47,XY,+12[12] |
| 19 | 46,XY,add(18)(p11)[14]/46,XY[4] |
| 20 | 46,XX,del(3)(p21),t(11;14)(q11;q32),inc[cp4]/46,XX[2] |
| 21 | 47,XY,+12[12]/47,XY,del(11)(q14q23),+12[2] |
| 22 | 47,XY,+12[10]/46,XY[3] |
| 23 | 47,XY,+12[13]/47,XY,del(6)(q23q25),+12[4] |
| 24 | 46,XX,add(3)(p25),add(9)(q?22)[4]/46,XX[7] |
| 25 | 46,XY,del(11)(q22q23)[3]/46,XY,del(11)(q22q23),del(13)(q12q14)[8] |
| 26 | 46,XY,del(11)(q22q23)[3]/46,XY,del(11)(q22q23),del(13)(q12q14)[2]/46,XY[3] |
| 27 | 46,XY,del(8)(p?21p?10),inv(9)(p13q21)c[12] |
| 28 | 46,XY,del(11)(q22q23)[5]/46,XY,del(6)(q25q27)[4]/46,XY,del(6)(q25q27),del(11)(q22q23)[3]/  46,XY[6] |
| 29 | 47,XX,+12[14] |
| 30 | 46,XX,del(11)(q22q23)[2]/46,XX,t(8;14)(q24;q21),del(11)(q22q23)[2]/46,XX,del(11)(q14q23)[3]/46,XX[8] |
| 31 | 47,XX,+12[7]/46,XX[3] |
| 32 | 46,XY;del(13)(q13q14)[10]/46,XY[2] |
| 33 | 46,XY,der(13)del(13)(q12q14)t(13;20)(q21;p11),der(20)t(13;20)(q21;p11)[8]/46,XY[4] |
|  | ***Complex karyotype present*** |
| 1 | 45,XY,der(8;17)(q10;q10)[4]/45,XY,add(6)(q27),der(8;17)(q10;q10)[5]/45~46,XY,add(Y)(p?11), der(8;17)(q10;q10),+mar[cp2]/45~46,XY,add(Y)(p?11),add(6)(q27),der(8;17)(q10;q10),add(16)(q12),+mar[cp5] |
| 2 | 46,XX,del(11)(q22q23)[8]46,XX,t(4;17)(p14;q21),del(11)(q22q23)[2]46,XX,add(1)(p10),t(1;20)(p10;p10),der(4)t(4;17)(p14;q21),del(11)(q22q23),der(?13)t(1;?13)(p22;?)[cp3]/46,XX[3] |
| 3 | 42,X,-Y,add(3)(p21),add(5)(q35),del(6)(q?21q?23),-8,der(12)t(9;12)(q34;p13),  der(14;15)(q10;q10),-15,add(17)(p11)[cp10] |
| 4 | 44,X,-Y,del(1)(q12),del(6)(q13q25),-13,der(17)t(1;17)(q12;p11),der(19)t(13;19)(q21;q13)[11] |
| 5 | 46,XY,del(17)(p11)[6]/45-46,XY,-4,der(7)t(4;7)(q21;p22),del(13)(q12q21),del(17)(p11),inc[cp8] |
| 6 | 46,XY,der(16)t(16;17)(q24;q21),-17,+mar[2]/46,XY,add(9)(p24),der(16)t(16;17)(q24;q21), -17,+mar[8] |
| 7 | 46,XY,t(2;9)(q13;q32),-3,+12,der(17)t(3;17)(q12;p11)[12] |
| 8 | 45,XX,der(4)t(3;4)(q11;p14),del(14)(q24q32),del(15)(q22),-17[7]/46,XX,der(4)t(3;4)(q11;p14), del(14)(q24q32),del(15)(q22),-17,+21[3] |
| 9 | 45,XX,+2, der(2;18)(p10;q10),del(4)(q21),-13,der(17)t(13;17)(q14;p11),der(19)(4q21->4qter::19p13->19qter)[12] |
| 10 | 45,XY,-6,+der(12)t(1;12)(p13;p13),der(17;21)(q10;q10),add(22)(p11),inc[cp8] |
| 11 | 46,XX,add(17)(p12)[8]/44,XX,der(4)?t(4;18)(p14;q11),-8,add(11)(q23),add(17)(p12),-18[5] |
| 12 | 47,XY,+12[6]/46,XY,t(1;9)(p34;q34),+12,-17[6]/45,XY,del(10)(q22q24),+12,-13,-17[3] |
| 13 | 45~46,XX,del(7)(q22q32),-8,-11,del(11)(q14q23),del(13)(q12q14),+mar1,+mar2,inc[cp8] |
| 14 | 43~46,XY,add(2)(p?25),del(6)(q?21q?23),add(11)(q?23),del(13)(q12q21),  der(14)?add(14),inc[cp10] |
| 15 | 44,XY,add(4)(p16),der(6)t(6;17)(q21;q11),-9,-17,der(18)t(9;18)(q13;q23)[5]/46,XY[6] |
| 16 | 45,XX,t(3;9)(p21;p?22),t(3;16)(q21;q24),-20[10] |
| 17 | 46,XY,-8,add(10)(q26),del(11)(q14q23),del(13)(q12q21),?del(14)(q24),+mar,inc[cp5] |
| 18 | 45~47,XX,del(7)(q32),add(9)(q34),+12,add(14)(p11)[cp15] |
| 19 | 37~46,XY,add(8)(p11),del(11)(q14q23),-13,16,-21,+2mar,inc[cp8] |
| 20 | 46,XY,del(11)(q14q23)[7]/46,XY,der(11)add(11)(p14)del(11)(q14q23)[2]/46,XY,der(1)ins(1;?)(p34;?),der(5)(q?),del(6)(q23q25),del(11)(q14q23),del(13)(q12q21)[cp4] |
| 21 | 45,XY,t(2;12)(q31;p13),del(11)(q22q23),del(12)(p12),del(13)(q22q23),-15,add(18)(q22)[cp6]/  46,XY,der(1)t(1;5)(q21;q13),t(2;12)(q31;p13),del(3)(p?22),del(5)(q13),del(11)(q22q23),  add(18)(q22)[4] |
| 22 | 46,XY,del(11)(q22q23)[2]/46,XY,der(9)t(9;11)(q34;q13),del(11)(q22q23),der(20)t(?9;20)(q21;p13)[5] |
| 23 | 46,XX,t(6;15)(q26;q21),inv(9)(p21q21)c,del(11)(q22q23)[9]/46,XX,t(6;15)(q26;q21),del(7)(q22q32),inv(9)(p21q21)c,del(11)(q22q23)[9] |
| 24 | 45,XX,+1,der(1;8)(q10;q10),der(1;9)(p10;q10),del(11)(q14q25),add(19)(q13)[8] |

Values within brackets denote the number of metaphases with karyotype; “cp” before the value within brackets indicates composite karyotype, which describes a summarization of clonal cells that share some cytogenetic characteristics. Karyotyping per ISCN 2013 guidelines (4).

**Table S3.** Demographic and baseline characteristics in patients with successful karyotyping

|  | **Idelalisib/rituximab** | | | | | **Placebo/rituximab** | | |
| --- | --- | --- | --- | --- | --- | --- | --- | --- |
|  | **CK-positive**  **N = 26** | | **CK- negative**  **N = 37** | | ***P*-value^c^** | **CK- positive**  **N = 24** | **CK-negative**  **N = 33** | ***P*-value^c^** |
| Male | 20 (76.9) | | 26 (70.3) | | 0.7738 | 15 (62.5) | 20 (60.6) | 1.0000 |
| Age, years, median (range) | 69 (58–84) | | 73 (48–90) | | 0.3901 | 69 (60–83) | 72 (60–92) | 0.0649 |
| Rai stages  0/I–II/III–IV^a^, % | 0/27/69 | | 0/32/60 | | 0.5868 | 0/17/75 | 3/27/67 | 0.7115 |
| Years since diagnosis, median | 7.7 | | 8.8 | | 0.7908 | 8.5 | 9.3 | 0.7711 |
| Prior therapies, median (range) | 3.5 (1–7) | | 3.0 (1–10) | | 0.8258 | 3.0 (1–6) | 3.0 (1–10) | 0.3353 |
| Cytopenia at baseline^b^ |  | |  | |  |  |  |  |
| Any grade  Grades 3–4 | 22 (84.6)  10 (38.5) | | 31 (83.8)  10 (27.0) | | 1.0000  0.4137 | 23 (95.8)  13 (54.2) | 29 (87.9)  14 (42.4) | 0.3851  0.4295 |
| Total CIRS score >6 | 22 (84.6) | | 31 (83.8) | | 1.0000 | 19 (79.2) | 29 (87.9) | 0.4705 |
| Estimated CrCl ≥30–<60 mL/min | 12 (46.2) | | 16 (43.2) | | 1.0000 | 6 (25.0) | 18 (54.5) | **0.0324** |
| **High-risk parameter** | |  | |  |  |  |  |  |
| Del(17p) and/or *TP53* mutation | 16 (61.5) | | 16 (43.2) | | 0.2028 | 15 (62.5) | 12 (36.4) | 0.0641 |
| Del(11q) | 9 (34.6) | | 10 (27.0) | | 0.5834 | 8 (33.3) | 7 (21.2) | 0.3685 |
| Unmutated IGHV | 22 (84.6) | | 32 (86.5) | | 1.0000 | 20 (83.3) | 29 (87.9) | 0.7093 |
| β2-microglobulin >4 mg/L | 25 (96.2) | | 30 (81.1) | | 0.1254 | 18 (75.0) | 28 (84.8) | 0.4988 |
| **CLL-IPI risk group** |  | | | |  |  | |  |
| Intermediate risk | 1 (3.8) | | 1 (2.7) | | 0.2874 | 1 (4.2) | 1 (3.0) | 0.1261 |
| High risk | 9 (34.6) | | 20 (54.1) | |  | 8 (33.3) | 19 (57.6) |  |
| Very high risk | 15 (57.7) | | 14 (37.8) | |  | 14 (58.3) | 12 (36.4) |  |

Data presented as n (%) unless otherwise noted.

^a^Stages at screening.

^b^Anemia, thrombocytopenia, and/or neutropenia.

^c^*P*-values are based on Fisher’s exact test (for categorical variables) and Kruskal-Wallis test (for median comparison of continuous variables).

CIRS, Cumulative Illness Rating Scale; CK, complex karyotype; CLL-IPI, Chronic Lymphocytic Leukemia-International Prognostic Index; CrCl, creatinine clearance; IGHV, immunoglobulin heavy-chain variable region gene.

**Table S4.** Demographic and baseline characteristic of patients who were successfully karyotyped vs not successfully karyotyped

|  | **Successfully karyotyped** | | | **Not successfully karyotyped** | |  |
| --- | --- | --- | --- | --- | --- | --- |
|  | **Idelalisib/**  **rituximab**  **N = 63** | **Placebo/**  **rituximab**  **N = 57** | | **Idelalisib/**  **rituximab**  **N = 47** | **Placebo/**  **rituximab**  **N = 53** |  |
| Male | 46 (72.0) | 35 (61.4) | | 30 (63.8) | 33 (62.3) |  |
| Age, years, median (range) | 72 (48–90) | 71 (60–92) | | 70 (58–87) | 71 (47–88) |  |
| Rai stages 0/I–II/III–IV^a^, % | 0/30/63 | 2/23/70 | | 0/32/64 | 0/32/58 |  |
| Years since diagnosis, median | 7.9 | 9.1 | | 7.7 | 8.5 |  |
| Prior therapies, median (range) | 3 (1–10) | 3 (1–10) | | 4 (1–12) | 3 (1–9) |  |
| Cytopenia at baseline^b^ | | |  |  |  |  |
| Any grade  Grades ≥3 | 53 (84.1)  20 (31.7) | 52 (91.2)  27 (47.4) | | 41 (87.2)  15 (31.9) | 45 (84.9)  16 (30.2) |  |
| Total CIRS score >6 | 53 (84.1) | 48 (84.2) | | 44 (93.6) | 43 (81.1) |  |
| Estimated CrCl ≥30–<60 mL/min | 28 (44.4) | 24 (42.1) | | 20 (42.6) | 15 (28.3) |  |
| **High-risk parameters** | | | |  | | |
| Del(17p) and/or*TP53* mutation | 32 (50.8) | 27 (47.4) | | 14 (29.8) | 22 (41.5) |  |
| Del(11q) | 19 (30.2) | 15 (26.3) | | 17 (36.2) | 17 (32.1) |  |
| Unmutated IGHV | 54 (85.7) | 49 (86.0) | | 37 (78.7) | 44 (83.0) |  |
| β2-microglobulin >4 mg/L | 55 (87.3) | 46 (80.7) | | 39 (83.0) | 39 (73.6) |  |
| **CLL-IPI risk group** | | | |  | | |
| Intermediate risk | 2 (3.2) | 2 (3.5) | | 2 (4.3) | 8 (15.1) |  |
| High risk | 29 (46.0) | 27 (47.4) | | 31 (66.0) | 19 (35.8) |  |
| Very high risk | 29 (46.0) | 26 (45.6) | | 12 (25.5) | 21 (39.6) |  |

Data presented as n (%) unless noted otherwise.

^a^Stages at screening.

^b^Anemia, thrombocytopenia, and/or neutropenia.

CIRS, Cumulative Illness Rating Scale; CLL-IPI, Chronic Lymphocytic Leukemia-International Prognostic Index; CrCl, creatinine clearance; IGHV, immunoglobulin heavy-chain variable region gene.

**Table S5.** Overall survival and progression-free survival in patients who were successfully karyotyped vs not successfully karyotyped

|  | **Idelalisib/rituximab** | | | **Placebo/rituximab** | | |
| --- | --- | --- | --- | --- | --- | --- |
|  | **Successfully karyotyped**  **N = 63** | **Not successfully karyotyped**  **N = 47** | | **Successfully karyotyped**  **N = 57** | | **Not successfully**  **karyotyped**  **N = 53** |
| OS, median (95% CI), months | 39.8 (28.3, 57.3) | | 47.4 (25.4, NR) | | 18.1 (11.1, 50.0) | 51.3 (16.6, NR) |
| Unadjusted HR (95% CI), *p*-value^a^ | 1.07 (0.61, 1.88); *p* = 0.8140 | | | | 1.64 (0.96, 2.78); *p* = 0.0655 | |
| PFS, median, (95% CI), months | 20.8 (16.4, 27.9) | | 20.3 (16.3, 32.4) | | 10.7 (5.7, 15.9) | 9.9 (5.6, 26.2) |
| Unadjusted HR (95% CI), *p*-value^a^ | 0.92 (0.56, 1.52); *p* = 0.7571 | | | | 1.21 (0.73, 1.99); *p* = 0.4659 | |

^a^Hazard ratio and 95% CIs are calculated using the Cox proportional hazards model without any adjustment, *p*-value from unstratified Log-Rank Test.

CI, confidence interval; HR, hazard ratio; NR, not reached; PFS, progression-free survival; OS, overall survival.

**Figure S1.** Consort diagram


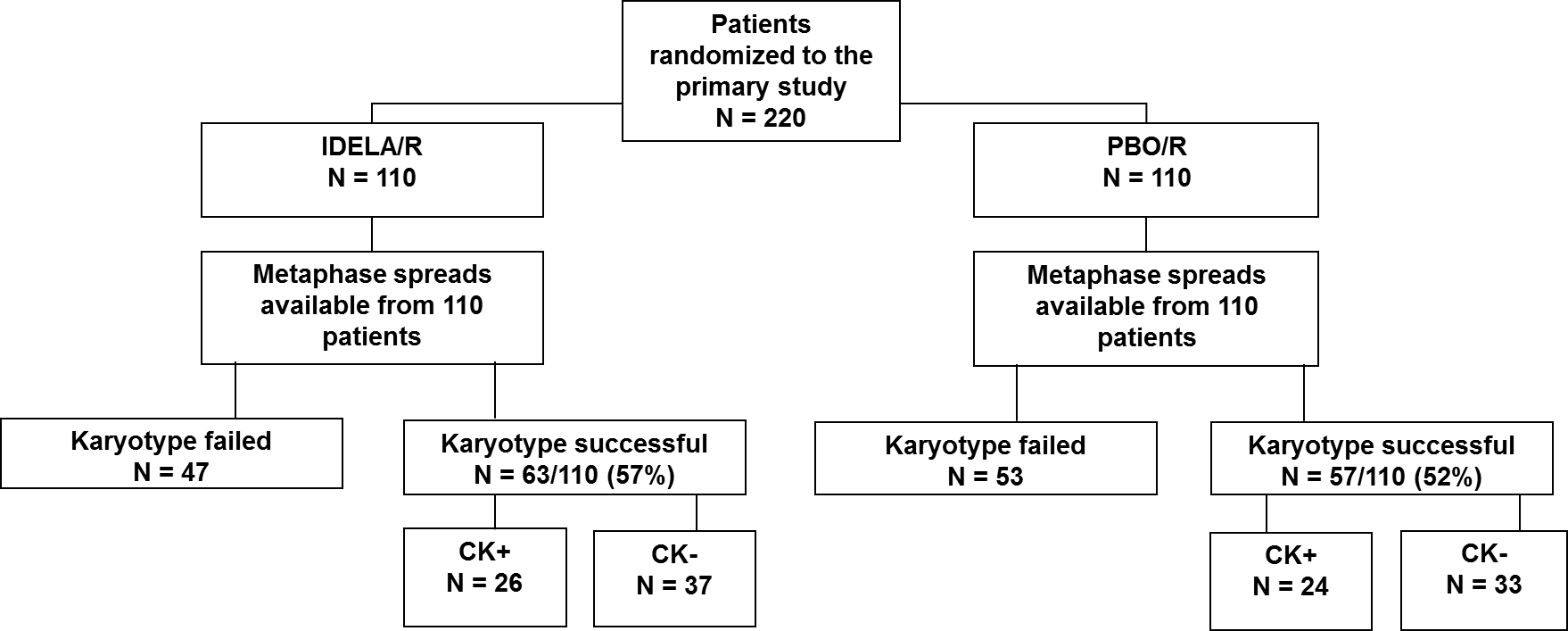


CK, complex karyotype; IDELA, idelalisib; PBO, placebo; R, rituximab.

**References**

1. Furman RR, Sharman JP, Coutre SE, Cheson BD, Pagel JM, Hillmen P*, et al.* Idelalisib and rituximab in relapsed chronic lymphocytic leukemia. *N Engl J Med* 2014 Mar 13; **370:** 997-1007.

2. Hallek M, Cheson BD, Catovsky D, Caligaris-Cappio F, Dighiero G, Dohner H*, et al.* Guidelines for the diagnosis and treatment of chronic lymphocytic leukemia: a report from the International Workshop on Chronic Lymphocytic Leukemia updating the National Cancer Institute-Working Group 1996 guidelines. *Blood* 2008 Jun 15; **111:** 5446-5456.

3. Brejcha M, Stoklasova M, Brychtova Y, Panovska A, Stepanovska K, Vankova G*, et al.* Clonal evolution in chronic lymphocytic leukemia detected by fluorescence in situ hybridization and conventional cytogenetics after stimulation with CpG oligonucleotides and interleukin-2: a prospective analysis. *Leuk Res* 2014 Feb; **38:** 170-175.

4. Shaffer LG, McGowan-Jordan J, Schmid M (eds). *ISCN (2013): An International System for Human Cytogenetic Nomenclature*. S. Karger: Basel, 2013.

5. Simons A, Shaffer LG, Hastings RJ. Cytogenetic Nomenclature: Changes in the ISCN 2013 Compared to the 2009 Edition. *Cytogenet Genome Res* 2013; **141:** 1-6.

6. Stilgenbauer S, Schnaiter A, Paschka P, Zenz T, Rossi M, Dohner K*, et al.* Gene mutations and treatment outcome in chronic lymphocytic leukemia: results from the CLL8 trial. *Blood* 2014 May 22; **123:** 3247-3254.

7. Cheson BD, Byrd JC, Rai KR, Kay NE, O'Brien SM, Flinn IW*, et al.* Novel targeted agents and the need to refine clinical end points in chronic lymphocytic leukemia. *J Clin Oncol* 2012 Aug 10; **30:** 2820-2822.
